# Induced Lactation in Non-gestational Mothers in Iran: Outcomes and Predictors of Breastfeeding Success

**DOI:** 10.34172/aim.33516

**Published:** 2025-03-01

**Authors:** Seyed Alireza Marandi, Nahid Ezzeddin Zanjani, Mahmood Ravari, Mojgan Mazloom, Ziba Mohammad Khanlou, Amir Kasaeian

**Affiliations:** ^1^Iranian Breastfeeding Promotion Society, Tehran, Iran; ^2^Liver and Pancreatobiliary Diseases Research Center, Digestive Diseases Research Institute, Shariati Hospital, Tehran University of Medical Sciences, Tehran, Iran; ^3^Digestive Oncology Research Center, Digestive Diseases Research Institute, Shariati Hospital, Tehran University of Medical Sciences, Tehran, Iran; ^4^Clinical Research Development Unit, Shariati Hospital, Tehran University of Medical Sciences, Tehran, Iran

**Keywords:** Breastfeeding success, Exclusive breastfeeding, Induced lactation, Milk production, Non-gestational mothers

## Abstract

**Background:**

Induced lactation allows non-gestational mothers, such as those adopting or using surrogacy, to breastfeed their children. While widely practiced in various contexts, little research exists on its application in Iran. This study aimed to assess the success and duration of induced lactation in Iranian mothers without prior pregnancy.

**Methods:**

This descriptive-analytical study was conducted between 2013 and 2021, recruiting mothers who became parents through surrogacy or adoption. Participants followed a protocol using hormonal (combined oral contraceptives, domperidone) and non-hormonal methods (breast stimulation). Data on breastfeeding duration, exclusivity, and reasons for cessation were collected through regular follow-up. Chi-square tests and multivariable logistic regression were used to assess factors influencing breastfeeding duration.

**Results:**

Of the 117 mothers, 100 breastfed for<180 days and 17 for≥180 days. The median breastfeeding duration was significantly longer in the≥180 days group (240 days vs. 30 days, *P*<0.001). Exclusive breastfeeding up to 120 days was much more common in the≥180 days group (70.60% vs. 24%, *P*<0.001) while biological reasons were the primary cause of cessation in both groups but with different frequencies (100% vs. 75%, *P*=0.022). The results of logistic regression showed that exclusive breastfeeding for 120 days increased the likelihood of successful breastfeeding for≥180 days (OR: 5.72 (1.76‒18.61), *P*=0.004) adjusting for mother’s age (1.02 (0.92‒1.12), *P*=0.755), duration of Domperidone use (1.01 (0.98‒1.04), *P*=0.536), duration of LD use (1.04 (0.95‒1.15), *P*=0.366), and reason for stopping breastfeeding (7.02 (0.39‒125.30), *P*=0.185).

**Conclusion:**

This first Iranian study on induced lactation underscores the role of early exclusive breastfeeding in extending breastfeeding duration. Pharmacological support aids in initiating lactation, but long-term success relies on consistent breastfeeding practices. These findings highlight the need for culturally tailored guidelines and support for non-gestational mothers in Iran.

## Introduction

 Today, the mothers who are unable to carry the infant *in utero* are extremely eager to breastfed their surrogacy or adopted infant by using the current protocols (to induced an artificial pregnancy) under their physician’s supervision. Therefore, they would be able to breastfed their infants similar to other mothers according to the AAP, WHO and UNICEF recommendation stated below:

 Exclusive breastfeeding for 6 months after birth and continue breastfeeding along with appropriate complementary foods introduced at about 6 months, as long as mutually desired by mother and child for two years or beyond.

 Induced lactation, the process of stimulating milk production in women who have not undergone pregnancy, has become an increasingly important topic, particularly for adoptive mothers and those using surrogacy. This process not only allows mothers to provide their children with the many known benefits of breastfeeding (including essential nutrition and immune support) but also strengthens the mother-infant bond, which is vital for emotional well-being and development. Typically, lactation is triggered by the hormonal shifts that occur during pregnancy, but with carefully designed medical interventions, this natural process can be initiated even without a history of pregnancy.^1-3^

 To induce lactation, a combination of hormonal and non-hormonal methods is often employed. Hormonal regimens frequently involve the use of combined oral contraceptives (COCs) to simulate pregnancy, followed by the administration of prolactin-stimulating drugs such as domperidone or metoclopramide to kickstart milk production.^4^ Alongside these pharmaceutical aids, physical breast stimulation through regular pumping or manual expression plays a crucial role in enhancing milk supply. This process has been well-documented in cases ranging from adoptive mothers to those with complex medical histories, such as breast cancer survivors, demonstrating that tailored approaches can overcome significant challenges​.^2,4,5^

 Despite the progress made in understanding and applying induced lactation, comprehensive, standardized guidelines are still lacking, particularly in specific cultural contexts such as Iran.^2,3^ This study aims to address the knowledge gap by evaluating the success rates, methodologies, and challenges faced by Iranian mothers—especially those who become parents through surrogacy or adoption. By investigating this underexplored area, the research hopes to provide data that can support the development of national guidelines and offer mothers in similar situations the opportunity to breastfeed their children.

## Materials and Methods

###  Ethical Considerations

 This study followed the ethical guidelines set by the Declaration of Helsinki.^6^ All participants provided informed consent after being fully briefed on the study’s objectives and procedures. Confidentiality was strictly maintained to protect personal data. Ethical approval was granted by the Iranian Breastfeeding Promotion Association review board, ensuring that participant safety and well-being were prioritized throughout the study, with the option for participants to withdraw at any point without any repercussions.

###  Study Design and Population

 This descriptive-analytical study was designed to explore the outcomes of induced lactation in mothers without a history of pregnancy, specifically those who became parents through surrogacy or adoption. The study was conducted between 2013 and 2021, recruiting participants from the Iranian Breastfeeding Promotion Association. Mothers who had sought guidance and support from the association, either through in-person visits or phone consultations, formed the study population. These mothers were all interested in inducing lactation to breastfeed their children, despite never having experienced pregnancy. The recruitment process included mothers from various socio-economic backgrounds, ensuring a broad representation of experiences and challenges.

###  Lactation Induction Protocol

 To initiate lactation, the included participants followed a protocol involving both pharmacological and non-pharmacological methods. Hormonal treatment played a key role, with participants receiving COCs to simulate the hormonal changes of pregnancy. This was followed by the use of Domperidone, a drug known to enhance prolactin levels, which stimulates milk production. Mothers were also instructed to begin regular breast pumping approximately 4‒6 weeks prior to the expected arrival of their infant, pumping 8 to 10 times daily using an electric pump. This protocol was adapted from the well-established Newman-Goldfarb method, widely used in lactation induction.^7^

 For mothers who opted for non-hormonal approaches due to personal preference or medical contraindications, lactation was induced through mechanical breast stimulation alone. After the arrival of their child, if the milk supply remained low, some mothers were offered the use of a Supplemental Nursing System (SNS) to encourage the infant’s suckling and further stimulate milk production.

###  Data Collection

 Data were collected from participant interviews and medical records. The gathered information included maternal age, method of lactation induction (hormonal vs. non-hormonal), duration of medication usage, breast pumping frequency, and duration of exclusive breastfeeding. Additionally, reasons for discontinuing breastfeeding were categorized into biological factors (such as insufficient milk supply or infant health issues) and human factors (such as family decisions or social support). Infant feeding outcomes were assessed through regular follow-up, which included monitoring breastfeeding duration and exclusivity. The study followed participants for up to 2 years post-induction, with visits scheduled every 2 months during the first 6 months and biannually thereafter.

###  Outcomes

 The primary outcome of the study was the total duration of breastfeeding, with a focus on mothers who continued breastfeeding beyond 180 days. Secondary outcomes included the success of lactation induction, defined as milk production for at least 30 days, and the duration of exclusive breastfeeding, where the infant received only breast milk for the first 120 days. The reasons for stopping breastfeeding were also evaluated to identify common challenges and barriers faced by the participants.

###  Statistical Analysis

 Descriptive statistics were used to summarize the data. Categorical variables were expressed as frequencies and percentages, while continuous variables were presented using medians and interquartile ranges (IQR). The Chi-square test or Fisher exact test was employed to assess the homogeneity between the surrogacy and adoption groups for categorical variables, and *t*-tests or Wilcoxon rank-sum tests were used for continuous variables. To further analyze the likelihood of breastfeeding continuation beyond 180 days, a multivariable penalized logistic regression model was applied, using the “firthlogit” command in Stata. We used penalized logistic regression for bias reduction in our small sample. Penalized logistic regression, particularly Firth’s method, significantly reduces bias in parameter estimates, especially in small sample sizes. Traditional maximum likelihood estimation can yield biased results when events are rare or when there is complete or quasi-complete separation in the data. By introducing a penalty term into the likelihood function, penalized methods stabilize estimates and enhance model reliability. Effective factors considered in the model included maternal age, duration of medication use (Domperidone and COCs), reasons for stopping breastfeeding (biological vs. human factors), and whether exclusive breastfeeding was maintained for the first 120 days. Results were presented as odds ratios (ORs) with 95% confidence intervals (CIs), and statistical significance was set at a two-sided *P* value ≤ 0.05. All analyses were conducted using STATA version 16.

## Results

 A total of 117 mothers participated in the study, with 100 breastfeeding for < 180 days and 17 breastfeeding for ≥ 180 days. A descriptive analysis of the results are presented in Table 1. The median breastfeeding duration for the group that continued breastfeeding for ≥ 180 days was significantly higher at 240 days (IQR 300‒425), compared to 30 days (IQR 60‒90) in the group that breastfed for < 180 days (*P* < 0.001). This difference highlights a substantial variation in breastfeeding duration between the two groups.

**Table 1:** Descriptive Analysis Table

**Variables**	**Subgroups**	**Count**	**Percent**
Mother's age (years)	< 30	39	29.3
	31‒35	38	28.6
	36‒40	33	24.8
	> 40	23	17.3
Cause of infertility	Miscarriage	27	20.3
	Uterine issues	40	30.1
	Rokitansky syndrome	19	14.3
	Failed IVF	13	9.8
	Husband's infertility issue	15	11.3
	Other	19	14.3
Milk induction method	Medication (COC and domperidone) + breast pumping	130	97.7
Duration of domperidone use	Less than 2 months	8	6.84
	2 months	34	29.05
	2.5‒3 months	37	31.62
	> 3 months	38	32.48
Duration of COC use	Not used	6	5.13
	Less than 30 days	16	13.68
	30 days	72	61.54
	> 30 days	23	19.66
Infant feeding with produced milk	No	16	12.0
	Yes	117	88.0
Exclusive feeding with produced milk	No	81	69.2
	Yes	36	30.8
Duration of feeding with produced milk (months)	< 1 month	24	20.51
	1 to 6 months	76	64.96
	6 to 12 months	11	9.40
	1 to 2 years	2	1.71
	> 2 years	4	3.42
Reasons for discontinuation of breastfeeding in 117 infants	Human reasons		
	Late arrival of infant	9	36
	Family decision	6	24
	Lack of physician support	10	40
	Biological reasons		
	Infant issues	6	6.52
	Maternal issues	12	13.04
	Completion of the breastfeeding period	4	4.35
	Low milk volume or perception of insufficient milk	25	27.18
	Infant refusal, flat nipples, and prematurity	45	48.91

 The analysis of exclusive breastfeeding during the first 120 days showed a marked disparity between the two groups. Among mothers who breastfed for ≥ 180 days, 70.59% maintained exclusive breastfeeding for the first 120 days, compared to only 24% in the group that breastfed for < 180 days (*P* < 0.001). These results suggest that early exclusive breastfeeding strongly predicts a longer breastfeeding duration (Table 2).

**Table 2:** Baseline Characteristics of Participants

		**Breastfeeding<180 Days**	**Breastfeeding≥180 Days**	***P***
Age, years		34.44 ± 6.23	36.00 ± 0.62	0.332
Duration of Domperidone use, days		75.35 ± 19.47	78.53 ± 1.95	0.531
Duration of LD use, days		30.47 ± 5.82	32.94 ± 0.60	0.128
Breastfeeding duration, days		60 [30‒90]	300 [240‒425]	< 0.001
Exclusive breastfeeding duration, days		7.0 [7.0‒14.0]	30.0 (10.5‒30.0)	0.064
Reason for stopping breastfeeding, %	Human factors	25 (25.0%)	0	0.022
	Biological factors	75 (75.0%)	17 (100%)	
Exclusive breastfeeding up to 120 days, %		24 (24.0%)	12 (70.6%)	< 0.001

Data are presented as mean ± standard deviation, median [interquartile range], and number (%). LD indicates low-dose birth control pills.

 The analysis of reasons for stopping breastfeeding revealed significant differences between the two groups. In the group that breastfed for < 180 days, 75% of the mothers cited biological factors as the primary reason for cessation, whereas all mothers who breastfed for ≥ 180 days reported biological reasons (*P* = 0.022). Notably, none of the mothers in the 180-day or longer group stopped breastfeeding due to human factors, further emphasizing the impact of biological challenges on breastfeeding cessation in the shorter-duration group.

 Multivariable logistic regression analysis showed that exclusive breastfeeding up to 120 days was the only factor significantly associated with extended breastfeeding duration ≥ 180 days (Table 3). Mothers who exclusively breastfed during this period were more than five times as likely to continue breastfeeding for ≥ 180 days (OR: 5.72, 95% CI: 1.76‒18.61, *P* = 0.004). Other factors, including maternal age and duration of medication use (domperidone and low doses (LD)), did not have a statistically significant effect on prolonged breastfeeding duration.

**Table 3:** Penalized Multivariable Logistic Regression Results for Factors Associated with Breastfeeding ≥ 180 Days

		**Odds Ratio (95% CI)**	***P***
Age, years		1.02 (0.92‒1.12)	0.755
Duration of Domperidone use, days		1.01 (0.98‒1.04)	0.536
Duration of LD use, days		1.04 (0.95‒1.15)	0.366
Reason for stopping breastfeeding	Human (reference)	Ref.	0.185
	Biological	7.02 (0.39‒125.30)	
Exclusive breastfeeding for up to 120 days		5.72 (1.76‒18.61)	0.004

LD indicates low-dose birth control pills.

## Discussion

 This study represents the first of its kind in Iran to evaluate induced lactation in non-gestational mothers, specifically those who became parents through surrogacy or adoption. The results offer valuable insights into the role of early exclusive breastfeeding in promoting extended breastfeeding durations. Our findings suggest that mothers who practice exclusive breastfeeding during the first 120 days are more likely to continue breastfeeding for ≥ 180 days, underscoring the importance of early lactation practices in the success of induced lactation. These results align with previous studies in the field, which demonstrate the crucial role of early and consistent breastfeeding efforts in maintaining lactation, even in complex cases such as mothers with prior medical conditions or those undergoing surrogacy.^4,5^

 Our findings are consistent with a rich body of literature that has explored various aspects of induced lactation and relactation. For example, Mead reported successful relactation practices in Papua New Guinea as early as 1935.^8^ Similarly, anthropological observations have documented milk production in women who had never experienced pregnancy, solely through regular infant suckling.^8^ In a previous study including 606 American and Canadian women, 366 mothers relactated, and 240 induced lactation for their adopted infants, achieving a success rate of 66%.^9^ Further, a study in Papua New Guinea reported a success rate of 89% among 27 mothers who adhered strictly to a lactation protocol, with 12 of these mothers having no prior breastfeeding experience.^10^ These findings reinforce the efficacy of structured protocols, such as the Newman-Goldfarb method, which combines pharmacological and non-pharmacological approaches.^10^ Additionally, randomized studies, such as one comparing breast stimulation alone versus stimulation combined with domperidone or metoclopramide, found a 92% complete success rate in relactation.^11^ Notably, case studies have demonstrated successful lactation even in highly challenging situations, such as a mother with a history of breast cancer, and co-lactation by both parents in a same-sex couple.^5^

 In comparison to current evidence, our findings reinforce the effectiveness of both pharmacological and non-pharmacological methods in supporting lactation induction. Similar to the case study of a biological mother after gestational surrogacy of twins, our research shows that medications like domperidone can support lactation, but it is early exclusive breastfeeding that significantly predicts long-term breastfeeding success.^4^ While pharmacological interventions can stimulate milk production, sustained lactation is more dependent on behavioral factors, such as frequent breastfeeding and early exclusivity, rather than the duration of medication use.

 In addition, the challenges identified in our study mirror those reported in other research on induced lactation. Biological factors, such as insufficient milk supply, were the most common reasons for stopping breastfeeding both in our study and others​.^5,10^ However, the absence of human factors, such as social or emotional reasons, in the group that breastfed for ≥ 180 days indicates that adequate support (both emotional and medical) can help overcome many of the biological challenges associated with lactation induction.^2^ This reinforces the need for structured support systems, including healthcare professionals and lactation consultants, to guide mothers through the process of induced lactation, particularly during the critical early stages.^1^

 Our study also highlights the need for further research on induced lactation in non-gestational mothers, particularly in cultural contexts like Iran, where this practice has been underexplored. Although our study provides valuable data, the absence of standardized protocols and tailored support programs for mothers in Iran limits the generalizability of the findings. Future research should focus on developing and implementing culturally sensitive protocols that address the unique challenges faced by non-gestational mothers in Iran, including those related to societal expectations and medical resources.

## Limitations and Their Impact on Generalizability of the Results

 Our study utilized a sample that may not fully represent the broader population due to its specific demographic characteristics or geographic location. This limitation may restrict the applicability of our findings to other populations or settings. Future research with more diverse samples is needed to validate our results across different demographics.

 The cross-sectional design of our study limits causal inferences. While associations can be identified, establishing direct cause-and-effect relationships is not feasible. This limitation suggests that while our findings may be relevant in the context studied, they should be interpreted with caution when applied to longitudinal scenarios or different contexts.

## Conclusion

 In conclusion, this study emphasizes the critical role of early exclusive breastfeeding in ensuring the success of induced lactation. While pharmacological interventions can be helpful, the key to sustaining breastfeeding lies in early, consistent efforts and adequate support systems. These findings can inform future protocols and healthcare strategies aimed at improving breastfeeding outcomes for non-gestational mothers, both in Iran and globally.

## References

[1] Meek JY, Noble L (2022). Policy statement: breastfeeding and the use of human milk. Pediatrics.

[2] Schnell A (2022). Successful co-lactation by a queer couple: a case study. J Hum Lact.

[3] Schnell A (2022). The Three Step Framework for Inducing LactationTM. J Hum Lact.

[4] Farhadi R, Philip RK (2017). Induction of lactation in the biological mother after gestational surrogacy of twins: a novel approach and review of literature. Breastfeed Med.

[5] Al-Mohsen ZA, Frookh Jamal H (2021). Induction of lactation after adoption in a Muslim mother with history of breast cancer: a case study. J Hum Lact.

[6] World Medical Association (2013). World Medical Association Declaration of Helsinki: ethical principles for medical research involving human subjects. Jama.

[7] Goldfarb L. Newman-Goldfarb Protocols for Induced Lactation: Decision Tool. Cincinnati, Ohio: Union Institute & University Doctoral Program; 2007.

[8] Lawrence RA, Lawrence RM. Breastfeeding: A Guide for the Medical Professional. Elsevier Health Sciences; 2021.

[9] Auerbach KG (1981). Extraordinary breast feeding: relactation/induced lactation. J Trop Pediatr.

[10] Cazorla-Ortiz G, Obregón-Guitérrez N, Rozas-Garcia MR, Goberna-Tricas J (2020). Methods and success factors of induced lactation: a scoping review. J Hum Lact.

[11] Shen Q, Khan KS, Du MC, Du WW, Ouyang YQ (2021). Efficacy and safety of domperidone and metoclopramide in breastfeeding: a systematic review and meta-analysis. Breastfeed Med.

